# The Anti-Ageing and Whitening Potential of a Cosmetic Serum Containing 3-*O*-ethyl-l-ascorbic Acid

**DOI:** 10.3390/life11050406

**Published:** 2021-04-29

**Authors:** Nicola Zerbinati, Sabrina Sommatis, Cristina Maccario, Serena Di Francesco, Maria Chiara Capillo, Raffaele Rauso, Martha Herrera, Pier Luca Bencini, Stefania Guida, Roberto Mocchi

**Affiliations:** 1 Department of Medicine and Surgery, University of Insubria, 21100 Varese, Italy; nicola.zerbinati@uninsubria.it; 2 Department of Molecular Medicine, UB-CARE S.r.l.-Spin-Off University of Pavia, 27100 Pavia, Italy; sabrina.sommatis@ub-careitaly.it (S.S.); cristina.maccario@ub-careitaly.it (C.M.); serena.difrancesco@ub-careitaly.it (S.D.F.); mariachiara.capillo@ub-careitaly.it (M.C.C.); 3 Maxillofacial Surgery Unit, University of Campania “Luigi Vanvitelli”, 80138 Naples, Italy; raffaele.rauso@unicampania.it; 4 Centro Avanzado de Dermatologia y Laser, San Pedro Sula 34101, Honduras; martha@dermahn.com; 5 Istituto di Chirurgia e Laser-Chirurgia in Dermatologia (I.C.L.I.D.), 20121 Milan, Italy; pl.bencini@iclid.it; 6 Dermatology Unit, Department of Surgical, Medical, Dental and Morphological Sciences Related to Transplant, Oncology and Regenerative Medicine, University of Modena and Reggio Emilia, 41124 Modena, Italy; stefania.guida@unimore.it

**Keywords:** melanin, skin ageing, reconstructed human epidermis, human keratinocytes, fibroblasts, photoprotection, vitamin C

## Abstract

Skin ageing has many manifestations such as wrinkles, dryness, hyperpigmentation, and uneven skin tone. Extrinsic and intrinsic factors, especially solar ultraviolet light (UVB), contribute to skin ageing; its main features are brown spots, alterations in melanin pigmentation, and a decrease in collagen and hyaluronic acid linked to oxidative stress. Several studies showed that topical products containing ingredients with antioxidant activity can reduce oxidative damage; to provide a maximum anti-ageing effect to the skin, topical products can combine various ingredients. C-SHOT SERUM contains a combination of two molecules with a proven anti-ageing activity: a high percentage (30%) of a more stable vitamin C derivative, 3-*O*-ethyl-l-ascorbic acid, and lactic acid (1%). The product showed a high biocompatibility, assessed through an MTT assay on keratinocytes and on Reconstructed Human Epidermis (RHE, SkinEthic); the anti-ageing activity was demonstrated on human dermal fibroblasts and keratinocytes by a statistically significant increase in collagen production and a reduction of a UVB-induced DNA damage marker (**γ**-H2AX histone), indicating DNA protection. Moreover, a depigmenting activity, shown by a highly significant decrease in melanin content on treated Reconstructed Human Pigmented Epidermis (RHPE), was assessed. According to the data of our study, the tested product contrasts the effect of skin ageing and irregular pigmentation due to the physiological decline of the skin.

## 1. Introduction

Skin ageing is characterized by intrinsic and extrinsic factors that cause impairment of skin integrity, structural degradation and alterations [1]. Intrinsic or chronological ageing is an uncontrollable process caused by a physiological decline due to passing years and is characterized by smooth, dry, pale, and finely wrinkled skin [2]. Extrinsic ageing is generally caused by environmental factors such as ultraviolet (UV) radiation from the sun (major cause of skin damage as photoageing), alcohol intake, poor nutrition, overeating, and environmental pollution [3,4]. UVA constitute 95% of the sun’s rays that reach the Earth, but exposure to UVB, the remaining 5%, has a much greater biological impact on the skin than exposure to UVA when comparing similar irradiation doses. A major part of UVB is adsorbed in the epidermis by DNA, aromatic amino acids of proteins, nicotinamide adenine dinucleotide (NADH), and nicotinamide adenine dinucleotide phosphate (NADPH), and does not penetrate deeper into the skin [5]. Regarding aged skin, brown spots also are found often as a consequence of alterations in the melanin pigmentation, particularly visible on the face and back of the hands [6]. Photoageing leads to a drastic decrease in skin hydration and total levels of collagen, elastin, and hyaluronic acid, causing a series of intracellular responses such as the production of oxidative stress. Particularly, UVB also can directly affect the DNA of skin cells through the formation of cyclobutane pyrimidine dimers (CPDs) and another dipyrimidine lesion, the (6–4) photoproducts [7].

Constant use of skin care products with added active molecules or antioxidants from an early age can lead to significant benefits in counteracting the effects of skin damage. The antioxidants, such as vitamins, have a preventative effect on ageing and their use, in the cosmetic field, is due to their ability to easily penetrate the skin thanks to their small molecular weight, their safeness, and their efficacy often found in the short term [8,9].

C-SHOT SERUM is a cosmetic product (Matex Lab SpA, Via Carlo Urbani 2, ang Enrico Fermi, Brindisi, Italy) containing a high concentration of vitamin C (30%), in particular the much more stable derivative 3-*O*-ethyl ascorbic acid (3OAA) and lactic acid (1%), which play a key role in many basic molecular anti-ageing-related processes like collagen stimulation, melanogenesis inhibition, and protection against photoageing and UV damage. The 3-*O*-ethyl ascorbic acid has an ethyl group at the third carbon position and this structural modification protects the 3-OH group from ionization and, thus, the molecule from oxidation [10].

Vitamin C has shown its anti-ageing activity already from use at 5% in cosmetic formulations; furthermore, to be effective, topical application requires pH levels below four to allow the delivery across the epidermal layer to be mediated by specific sodium-dependent vitamin C transporters (SVCT1 and SVCT2). Both transporters are hydrophobic membrane proteins that co-transport sodium, driving the uptake of vitamin C; SVCT1 is a low affinity transporter closed to epithelial cells in the small intestine and kidney, while SVCT2 is a high affinity transporter ubiquitous in the cells of the body. The vitamin C delivery is finely regulated by the availability of the SVCTs on the plasma membrane and, following its uptake, it is involved in many molecular processes. Several evidence demonstrate the role of vitamin C as a co-factor for the regulatory proline and lysine hydroxylases that stabilize the collagen molecule tertiary structure through the hypoxia-inducible factor (HIF)-1 activation, controlling the gene expressions involved in collagen synthesis (Col-1 and Col-3) and tissue remodeling as collagenases [11,12,13,14,15,16]. Vitamin C is a water-soluble antioxidant that protects the skin from reactive oxygen species (ROS) by solar radiation or environmental factors such as pollution [14]. Other studies evidenced vitamin C’s ability to interfere with the action of tyrosinase, the rate-limiting enzyme in melanogenesis. Particularly, it reduces the ortho-quinones obtained from the dihydroxyphenylalanine (DOPA) oxidation derived from tyrosinase catalysis. Indeed, it has been used in cosmetic products also to make the skin more radiant and brighter because it is able to act against the hyperpigmentation [15,16,17].

However, the physicochemical properties of the compound, such as rapid oxidation in solutions upon exposure to air, the melting point (190–192 °C) partition coefficient (log P_(o/w)_ = −1.85) and dissociation constant (pK_a_ = 4.25) reduce its delivery across the skin and its molecular efficacy. To overcome these disadvantages and ensure the molecular efficacy, several vitamin C derivatives were synthetized and evaluated for their potential as pro-ascorbic acid derivatives. Among these, 3OAA is more lipophilic than the original, hence exhibiting an improved permeation into the skin, demonstrating an efficient transdermal activity, and allowing the molecular mechanisms associated to the original compound (vitamin C) previously described. Overcoming the absorption limits due to the topical application, the choice of using 3OAA within cosmetic formulations has proved to be a valid means to improve the effectiveness of the original functional compound [18,19].

Besides vitamin C and its derivatives, topical α-hydroxy acids (α-HA) also showed clinical efficacy in the anti-ageing skin treatment. The Cosmetic Ingredient Review (CIR) Panel assessed the evidence that α-HA ingredients are non-toxic, mutagenic, or carcinogenic recommending a maximum concentration of 10% with a pH above 3.5 in a cosmetic formulation. Among α-HA, glycolic and lactic acid have been shown to be effective in glycosaminoglycan (GAG) and collagen stimulation but, also, in the treatment of various types of hyper-pigmentary lesions, such as photoageing, solar lentigines, and post-inflammatory hyperpigmentation. Specifically, the proposed molecular mechanism underlying the skin pigmentation resulting from UV exposure is the increased epidermal turnover generating skin exfoliation/peeling and the suppression of melanin synthesis by inhibiting tyrosinase activity resulting in shiny and taut skin [19,20,21].

During this study, we investigated the potential of a cosmetic product containing concentrated 3-*O*-ethyl ascorbic acid at 30% in combination with lactic acid at 1%, to contrast the effect of skin ageing and irregular pigmentation due to the normal physiological decline of the skin on both in vitro cell cultures and 3D reconstructed tissue models.

## 2. Materials and Methods

### 2.1. International Nomenclature of Cosmetic Ingredients (INCI)

The functional classification of ingredients contained in the tested serum is summarized in Table 1.

### 2.2. Cell Cultures

Human keratinocytes (HaCaT, BS code CL 168), were provided by I.Z.L.E.R. (Institute Zooprofilattico della Lombardia e Emilia Romagna) while human dermal fibroblasts (NHDF-Ad−Human Dermal Fibroblasts, Adult, code CC-2511) were provided by Lonza (Basel, Switzerland). Cell lines were grown in a complete medium constituted by Dulbecco’s Modified Eagle’s Medium (DMEM, Biowest, Nuaillé, France) High Glucose with 10% fetal bovine serum (FBS, Gibco-Fisher Scientific, Waltham, MA, USA), 1% of L-glutamine (Capricorn Scientific, Ebsdorfergrund, Germany), and antibiotics 100 U/mL penicillin and 100 μg/mL streptomycin (Capricorn Scientific, Ebsdorfergrund, Germany), in conditions of complete sterility and maintained in incubation at 37 °C with a 5% carbon dioxide (CO_2_) atmosphere.

The Reconstructed Human Epidermis (RHE) model (EPISKIN Laboratories, Lyon, France) was supplied by SkinEthic™ laboratories; it is a reconstructed tissue from normal human keratinocytes grown for 17 days in a chemically defined medium. The tissue model consists of a fully differentiated epidermis including a basal cell layer, stratum spinosum, stratum granulosum, and stratum corneum on a 0.5 cm^2^ surface of inert polycarbonate filter at the air–liquid interface [22,23,24].

The SkinEthic^TM^ Reconstructed Human Pigmented Epidermis (RHPE) model (EPISKIN Laboratories, Lyon, France) consists of a stratified and differentiated epidermal layer containing human melanocytes and keratinocytes. The cell model used is made of inert polycarbonate inserts of 0.5 cm^2^ on which normal human cells differentiate to form a cellular multilayer.

Regarding both reconstructed models (RHE and RHPE), two specific media provided by EPISKIN were used: maintenance and growth media (Table 2).

### 2.3. Biocompatibility

#### 2.3.1. Cell Viability (MTT Test)

Cell viability was evaluated using the 3-[4,5-dimethylthiazol-2-yl]-2,5 diphenyl tetrazolium bromide assay, as previously described [25]. Briefly, keratinocytes were homogeneously seeded in 96-well plates at a density of 1.5 × 10^4^ and incubated at 37 °C, with a 5% CO_2_ humidified atmosphere. After 24 h, cells were treated with the cosmetic product, starting at 40 mg/mL, following serial dilution (1:2) in cell medium (tested range 0.313–40 mg/mL). Untreated cells were used as the control (Ctrl). The test was conducted in three replicates for each dilution. After 24 h of treatment, cells were incubated with 1 mg/mL MTT solution (Merck, Darmstadt, Germany) at 37 °C for 2 h. After removing the medium from each well, isopropanol was added to dissolve formazan crystals and the absorbance was read at a 570 nm wavelength using a microplate reader (MultiSkan, Thermo Scientific, Waltham, MA, USA). Cell survival was calculated measuring the difference in optical density (OD) of the tested cosmetic product with respect to the control (Equation (1)).

Cell viability (%) = (OD_570nm_ test product/OD_570nm_ control) × 100 (1)

Reduction of cell viability by more than 30% is considered a cytotoxic effect.

#### 2.3.2. Skin Irritation Test on 3D Model

The day of receipt, RHE inserts were placed in the maintenance medium (6-well plate) under sterile conditions and stored in an incubator at 37 °C, at 5% CO_2_ overnight. After this pre-incubation step, the product was topically applied on the surface of the epithelium insert for 42 min at a 32 μL/cm^2^ concentration, thus entering into contact with the in vitro reconstructed epithelium directly. To parallel, the other inserts were treated with a negative control Dulbecco Phosphate Buffer Solution (DPBS, Merck, Darmstadt, Germany) and a positive control consisting of a 5% (*w*/*v* in water) solution of Sodium Dodecyl Sulfate (SDS, Merck, Darmstadt, Germany), representing the irritating treatment after a short exposure. Occurring at the end of the exposure step, RHE inserts were rinsed with DPBS and transferred in 6-well plates for incubation at 37 °C, 5% CO_2_, 95% humidified atmosphere for 42 h. Tissue viability was assessed using an MTT test: tissues were incubated with an MTT solution (1 mg/mL) for 3 h, then the extraction with isopropanol was performed. After extraction, the OD of the samples was quantified by spectrophotometry at a 570 nm wavelength.

Isopropanol was used as a blank. After subtracting the blank OD from all raw data, mean OD values and standard deviations (SD) were calculated. Viability of the epidermis treated with the product was calculated as a ratio of the corrected optical densities of the sample over the negative control (untreated sample) (Equation (2)).

Cell viability (%) = (OD_570nm_ test product/OD_570nm_ negative control) × 100 (2)

When the cell viability value was ≤50%, the product was classified as an irritant.

To better evaluate the skin irritation effect of the cosmetic product, as well as the viability resulting from the direct contact of the product on the inserts, the levels of interleukin (IL)-1α released after treatment also were measured after 42 h of recovery time by an Enzyme-linked Immunosorbent Assay (ELISA) kit (Diaclone, Besançon cedex, France) following the manufacturer’s protocol. Briefly, supernatants were collected and used for the coating of a specifically pre-treated 96-well ELISA plate provided in the kit. The standards were used to perform the standard curve (3.9–250 pg/mL). Samples, blank, and standards were added to each well in duplicate and the assay was performed according to the supplier’s instructions. The absorbance was then measured at 450 nm using a microplate reader (MultiSkan, Thermo Scientific, Waltham, MA, USA). Data were analyzed as mean ± SD.

### 2.4. Skin-Ageing

#### 2.4.1. Photoprotective Effect 

HaCaT cells were seeded (5 × 10^4^) homogeneously in 22 × 22 mm slides placed in Petri dishes and subsequently treated with the product at the highest non-cytotoxic concentrations (5–10 mg/mL). After 24 h, treatment was removed and cells were exposed to ultraviolet (UV)B at 2.5 mJ/cm^2^ (CAMAG^®^ UV Lamp 4, wavelength 302 nm, Muttenz, CH) as a dose capable to cause damage on a DNA level. The dose was measured using a Spectroline DRC-100X digital radiometer (Spectronics Corporation Westbury, NY, USA). After irradiation, cells were maintained in a complete medium for a recovery time (24 h) at 37 °C before fixation in methanol. The day after, samples were incubated with the anti-phospho-Histone γ-H_2_A.X (diluted 1:5000 in a DPBS buffer containing 1% BSA) primary antibody for 1 h. After washing, incubation with a secondary antibody diluted 1:200 was performed for 30 min; then, Hoechst 33258 staining to highlight the cells’ DNA followed. Images of fixed cells were taken with a Nikon Eclipse E400 fluorescence microscope equipped with a Canon Power Shot A590 IS digital camera. Regarding processing, approximately 200 total cells were counted for each slide (marked with Hoechst), and the positive ones for histone γ-H2AX were evaluated among these too.

#### 2.4.2. Collagen Synthesis

NHDF cells were homogeneously seeded in 24-well plates at a density of 6 × 10^4^ cells per well and incubated at 37 °C, with a 5% CO_2_ humidified atmosphere. After 24 h, the medium was replaced by one with a low FBS concentration (0.5%) as recommended in the kit. After 24 h, the two concentrations of the product (1.25 and 2.5 mg/mL), demonstrated to be non-cytotoxic and having the best solubility from the preliminary MTT so were chosen to be used in this assay (data not shown). Occurring at the end of the treatment, the supernatant of each sample was collected into a sterile tube and incubated overnight with the Isolation and Concentration Reagent (polyethylene glycol TRIS-HCl buffer, pH 7.6). The day after, the measurement of collagen synthesis was performed using a commercial kit (Sircol, Soluble Collagen assay kit, Biocolor Life Science Assays, Carrickfergus, UK), according to the manufacturer’s instructions, and the absorbance of samples was measured at 555 nm. The concentration of collagen then was calculated using a standard curve.

### 2.5. Depigmenting Effect

The day of receipt, RHPE inserts were placed in a maintenance medium (6-well plate) under sterile conditions, removing the excess of agar, and stored in an incubator at 37 °C, 5% CO_2_ overnight. After this pre-incubation step, the product was deposed daily on the surface of the stratum corneum of the tanned epidermal tissue (1 μL/insert) for four days, while the medium was replaced every day. DPBS was used as a negative control following the same protocol. Inserts were incubated at 37 °C, 5% CO_2_, 95% humidified atmosphere and, after 6 days (48 h after the last day of treatment), tissues were assessed for cell viability (MTT test) and melanin content.

#### 2.5.1. Cytotoxicity on RHPE (MTT Test)

RHPE tissue samples were incubated in an MTT solution (1 mg/mL) for 3 h at 37 °C, 5% CO_2_ and, subsequently, formazan crystals were dissolved in 1.5 mL isopropanol for 2 h at room temperature under agitation. Two thousand microliters were transferred in triplicate into a 96-well plate and the concentration of formazan was quantified by measuring the OD through spectrophotometry at a 570 nm wavelength. Cellular viability was obtained by comparing the OD of the insert treated with the cosmetic product and the OD of the negative controls expressed as a percentage.

#### 2.5.2. Melanin Content

Regarding melanin content, epidermal tanned tissues were removed from the insert by cutting out the polycarbonate filter and then plunged into 400 μL of Solvable Solution (Perkin Elmer, Waltham, MA, USA). Then, samples were heated at 100 °C for 45 min, centrifuged, and melanin extract was measured by spectrophotometry at a 500 nm wavelength using a synthetic melanin calibration curve as reference.

### 2.6. Statistical Analysis

All the experiments were conducted in triplicate, except when otherwise specified, and the results were expressed as a mean ± SD. Statistical significance was calculated using the One-Way analysis of variance (ANOVA) with Fisher’s Least Significant Difference (LSD) multiple comparison as the post-test. Statistical analysis was performed using GraphPad Prism version 9.0.0. (GraphPad Software, Inc., San Diego, CA, USA). Only for the melanin amount evaluation was the statistical significance calculated using the Student’s *t*-test.

## 3. Results

### 3.1. Evaluation of Cell Viability and Skin Irritation

**Figure 1:**
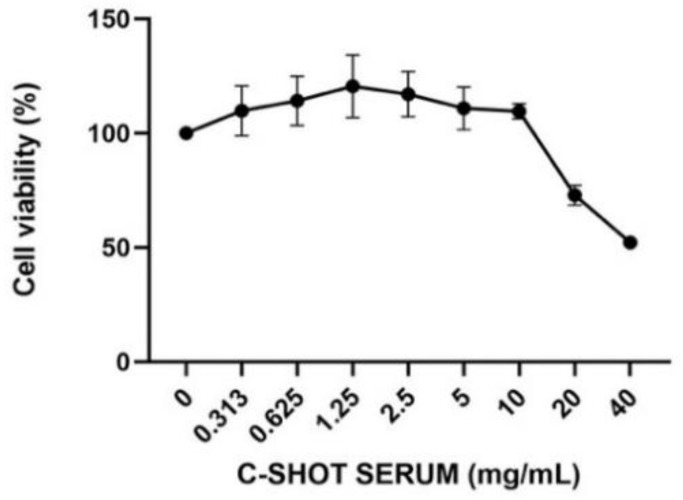
Cell viability after treatment with the product on HaCaT cells. Cells were treated with different concentrations (0.313–40 mg/mL) of the serum for 24 h, and cell viability was assessed through an MTT assay (*n* = 3, replicates = 3)**.**

The cosmetic product (Matex Lab SpA, Via Carlo Urbani 2, ang Enrico Fermi, Brindisi, Italy), showed high biocompatibility on human skin. The cytotoxicity test was required to evaluate the effect of the product on the cellular viability and to identify that the appropriate concentrations did not cause a decrease in cell respiration exceeding 30%. Regarding Figure 1 and Table 3, results of the HaCaT viability after treatment with different concentrations of the tested product (24 h) are shown, expressed as a percentage compared to the control (untreated cells). The cosmetic product showed cytotoxic activity only after treatment with the highest tested concentration, equal to 40 mg/mL, providing a cell viability percentage of 52.18%; higher product concentrations were not tested, assuming a further decrease in cell viability.

**Figure 2:**
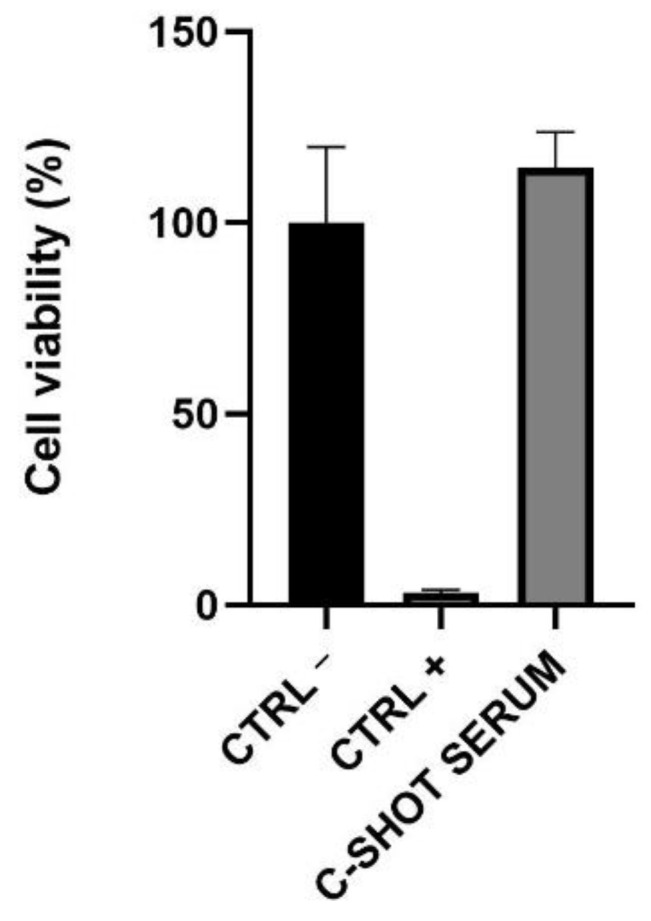
Cell viability on Reconstructed Human Epidermis (RHE). The inserts were treated with negative (Ctrl −, DPBS), positive (Ctrl +, SDS 5%) controls, and with the serum for 42 min. After 42 h of recovery, cell viability was assessed through an MTT assay.

Skin irritation was investigated on 3D-reconstructed human epidermis, evaluating tissue viability by an MTT test and the interleukin (IL)-1α amount. Reported in Figure 2 and in Table 4, the tested product did not show any irritant activity after a short direct exposure on the tested tissue with a viability greater than 50% (threshold used to identify a substance as not an irritant according to ISO 10993-10:2010 [26]).

**Figure 3:**
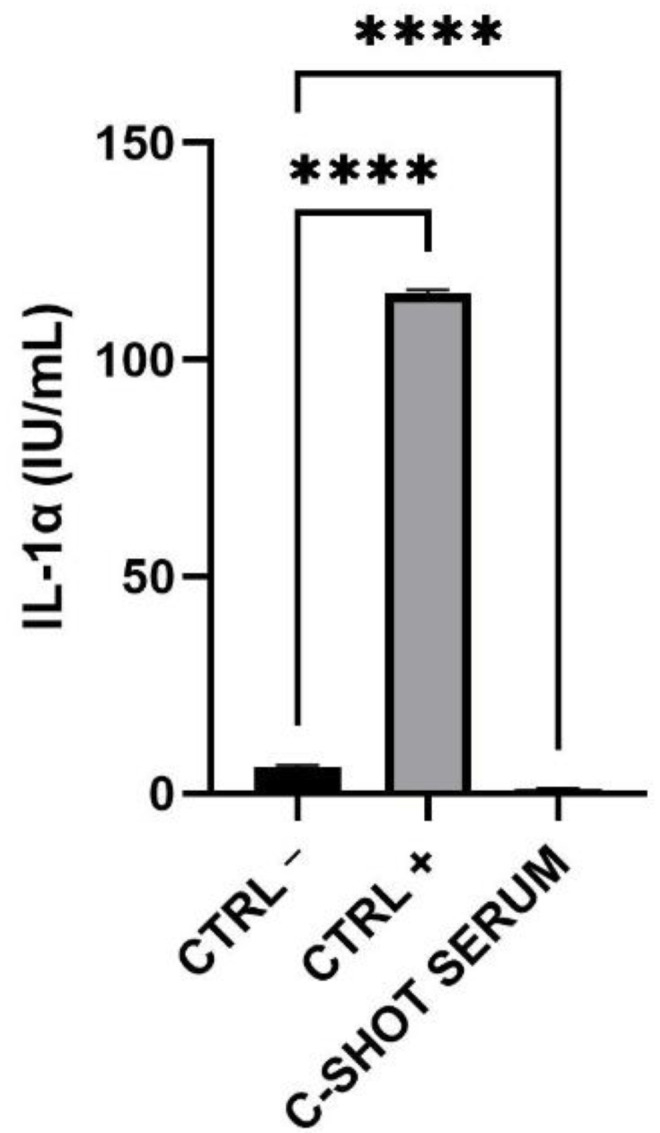
Expression of IL-1α (IU/mL) after treatment and 42 h of recovery of the RHE inserts with the negative (Ctrl −), positive (Ctrl +) controls and the serum. (*n* = 1, replicates = 4) **** *p* values ≤ 0.0001 were considered statistically significant compared to Ctrl −.

The amount of IL-1α released in the medium after treatment with the tested product was lower than the 9 International Unit (IU)/mL, threshold according to ISO 10993-10:2010 [26] (Figure 3 and Table 5), indicating a good biocompatibility of the product.

### 3.2. Protection Against Ultraviolet (UV)B-Induced DNA Damage

**Figure 4:**
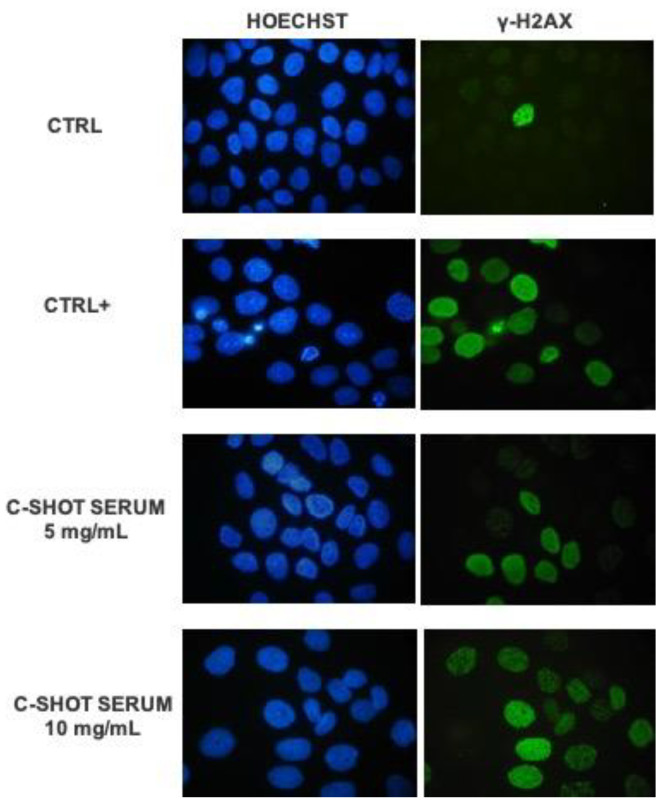
Representative fluorescence microscopy images (100×) of HaCaT cells after treatment with the product (5–10 mg/mL) and UVB 2.5 mJ/cm^2^ irradiation, the control sample (Ctrl, untreated, and non-irradiated cells) and positive control sample (Ctrl+, untreated, and irradiated cells). DNA is marked with Hoechst 33258 (blue staining), while green staining evidenced the phosphorylated histone γH2AX.

**Figure 5:**
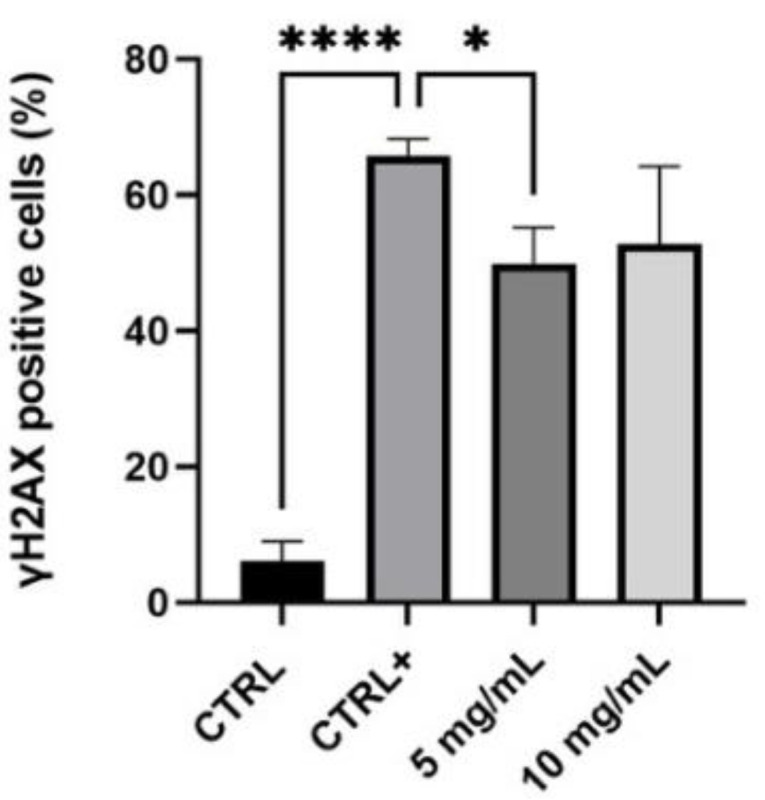
Quantification of γ-H2AX positive cells in control cells (Ctrl, untreated, and non-irradiated), positive control (Ctrl +, untreated, and irradiated cells) and in samples treated with the serum (5–10 mg/mL) (*n* = 2, replicates = 2) * *p* values ≤ 0.05 were considered statistically significant compared to positive control cells; **** *p* values ≤ 0.0001 were considered statistically significant compared to untreated cells.

The photo-protective activity of the product after UVB irradiation was investigated through the activation of phosphorylated H2AX histone (γ-H2AX). Considering this assay, the two highest concentrations with a cell viability greater than 80% were selected: 5 and 10 mg/mL. Figure 4 shows representative images of human keratinocytes (HaCaT) in fluorescence microscopy (magnification 100×) after treatment with the tested product while, in Figure 5 and in Table 6, the quantification of γ-H2AX positive cells is reported.

The obtained data highlighted that UVB radiations are a good stimulus to induce the activation of H2AX histone: there was a significant difference between untreated and non-irradiated cells (Ctrl) and the positive control, untreated, and UV-irradiated cells (Ctrl +) (*p* value **** ≤ 0.0001). Particularly, in the control sample the percentage of γ-H2AX positive cells was 6.15%, while the positive control was equal to 65.70%, indicating the efficacy of UVB stimulus. The lower tested concentration of the serum, 5 mg/mL, determined a statistically significant reduction of γ-H2AX positive cells compared to the positive control (*p* value * ≤ 0.05), equal to 24%, suggesting a protective effect against UV-damage. There seems to be a reduction trend even with the higher concentration, 10 mg/mL, but it does not have a statistically significant result.

### 3.3. Modulation of Collagen Synthesis

**Figure 6:**
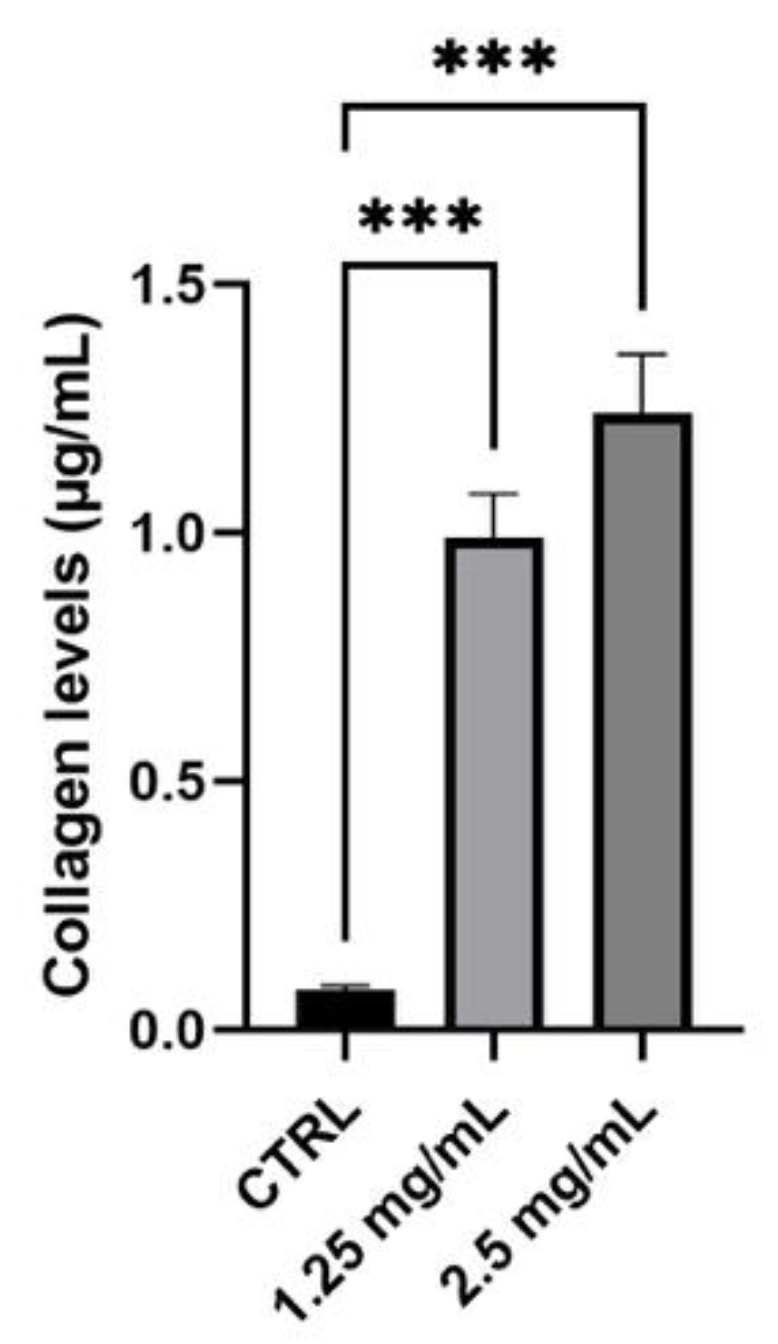
Collagen levels (μg/mL) in a sample treated with the product compared to the control (Ctrl, untreated cells) (*n* = 3, replicates = 2). *** *p* values ≤ 0.001 were considered statistically significant compared to the control (untreated cells).

Collagen synthesis was quantified to evaluate anti-ageing cosmetic potential. To perform this assay, human dermal fibroblasts (NHDF) were used and 1.25 and 2.5 mg/mL were chosen as the highest non-cytotoxic concentrations with the best solubility in the medium. Considering Figure 6 and Table 7, graphic and data are reported as a concentration (μg/mL) with respect to untreated cells (Ctrl) on the NHDF cell line. A pre-treatment of 24 h with the tested serum induced a significant increase in collagen production of more than 10 times with both tested concentrations, confirming the excellent capacity of this cosmetic serum to contrast the natural and physiological skin-ageing effect.

### 3.4. Evaluation of Melanin Content on Reconstructed Human Pigmented Epidermis (RHPE)

**Figure 7:**
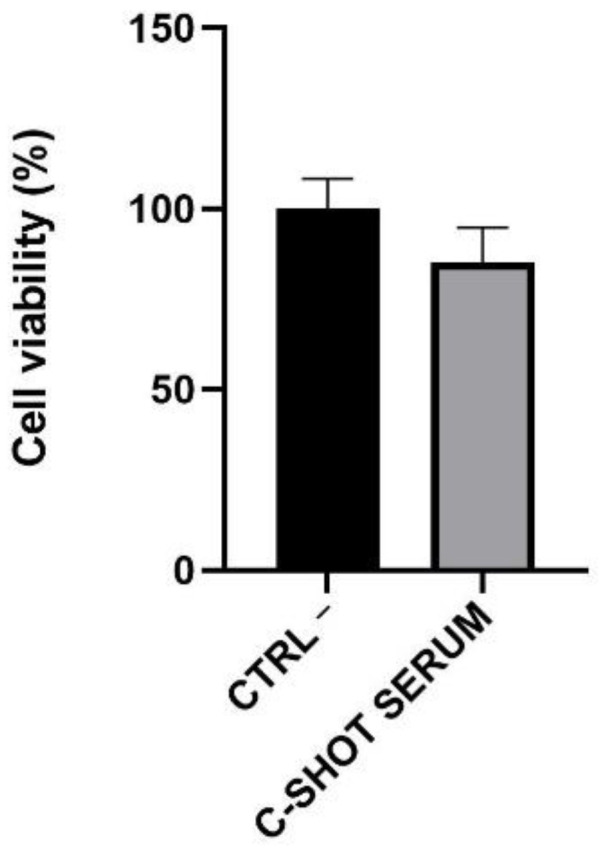
Cell viability expressed as a percentage compared to negative control (Ctrl −, cells treated with DPBS) following daily treatment (4 days) of the RHPE inserts with the serum (*n* = 1, replicates = 4).

The depigmenting activity was tested on the RHPE model on which the viability and content of extracted melanin, after repeated treatment of 4 days with the tested product, was investigated. Considering the obtained results by the analysis of tissue viability, the serum did not show any negative effect on viability, with a percentage greater than 80% compared to the negative control (Ctrl −), treated with Dulbecco Phosphate Buffer Saline (DPBS) (Figure 7). Even in the case with the RHE, 50% was the threshold above which viability was acceptable, according to ISO 10993-10:2010 [26].

**Figure 8:**
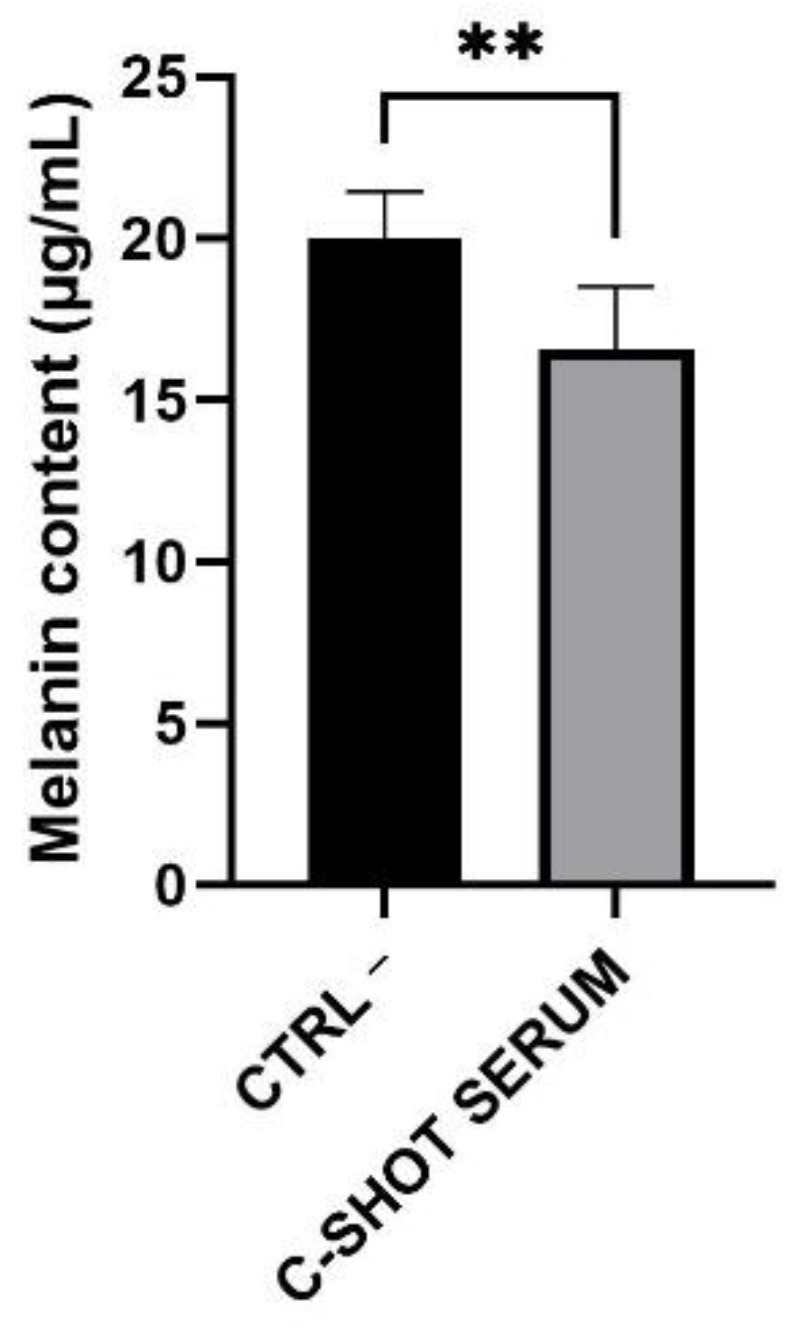
Melanin content (μg/mL) evaluated after four days of serum treatment of the tanned epidermis inserts compared to the untreated ones (negative control, Ctrl −) (*n* = 1, replicates = 4). ** *p* values ≤ 0.01 were considered statistically significant compared to untreated cells.

Melanin content was quantified and data are reported in Figure 8 and Table 8 as melanin content in µg/mL compared to the negative control (Ctrl −). The melanin amount in the control inserts was equal to 20.02 μg/mL, while in treated inserts it was reduced to 16.60 μg/mL, with a highly significant decrease equal to 17.10%. Statistical analysis was calculated using the Student’s *t*-test.

## 4. Discussion

Improvement in life expectancy results in increased functional cosmetic production, with the purpose of preventing the signs of ageing and keeping a healthy appearance. Skin-ageing is associated with deep wrinkles, loss of skin tone and elasticity, irregular pigmentation, and reduction in the thickness of the epidermis and dermis. Taking a molecular point of view, ageing is due to cumulative internal and external, structural and physiological alterations via genetics, light radiation exposure, marked loss of fibrillin-positive structures, and a reduced content of collagen fibers. Fibroblasts, which are the typical cells of the dermis, provide tensile strength and elasticity through the production and secretion of various components of the extracellular matrix (ECM). Collagen is one of these main components and is also the most abundant protein within the body, able to provide not only tensile strength but also cell adhesion and migration. The molecular knowledge of these biological events allows the formulation of functional innovative cosmetic products active on these, promoting an anti-ageing effect.

During this study, we investigated the cosmetic C-SHOT SERUM’s (Matex Lab SpA, Via Carlo Urbani 2, ang Enrico Fermi, Brindisi, Italy) ability to prevent skin-ageing through in vitro tests and 3D cell culture systems. Most in vitro models are based on a monolayer of one kind of cell line. This system shows some disadvantages because, in the in vivo environment, different cell types interact with each other within the extracellular matrix (ECM). It may be difficult to assess the actual activity of a substance only based on a cell monolayer without considering the complexity of a biological model such as the human skin. However, combining different assays can improve the effectiveness of in vitro models, and they could be a good and cost-efficient starting point. Moreover, to support the 2D model analyses performed, we decided to test the product also using an innovative system like 3D cell cultures, which represent a much more accurate microenvironment allowing us to mimic more closely the in vivo skin tissue [27,28].

The serum contains a high concentration of vitamin C, in particular its derivative 3-*O*-ethyl-l-ascorbic acid (3OAA) (30%) and lactic acid (1%), with a pH equal to 3.82 that allows good penetration and efficacy of these functional ingredients [17,20]. Since, with ageing, the pH of the skin increases to around 6.0, skin care products with a low pH in the range of 3.5–4.0 normalizes the epidermal barrier function [29]. Biocompatibility is the first property of a cosmetic that must be evaluated. During our study, it was assessed through a conventional in vitro assay (MTT test) and with a 3D model to study the skin irritation potential on a model closer to in vivo skin tissue. The serum showed an excellent biocompatibility, the starting point for evaluating further beneficial effects of the formulation. Vitamin C, contained in the product as a functional ingredient, is an antioxidant biomolecule that protects skin cells from oxidative damage after exposure to ultraviolet (UV) rays; it shows a proven skin anti-ageing effect for its ability to induce the production of collagen (Col)-1 and Col-3 (enhancer of collagen production), and the inhibition the collagenase 1 [13,16].

Extrinsic factors, especially UV radiation, are responsible for most age-related changes in skin appearance, producing free radicals, degrading collagen and elastin fibers, and inducing DNA damage. Therefore, the photo-protective activity from UVB rays and collagen synthesis stimulation were analyzed. The effect of UVB rays on the DNA damage of skin cells was evaluated in association with a pre-treatment of the product to investigate a photoprotective effect. The phosphorylation of histone H2AX (γ-H2AX) is a direct index of DNA molecular lesion [30] and it plays an important role in the DNA repair process. Therefore, damage to the keratinocytes’ DNA was determined by an immunofluorescence analysis of γ-H2AX positive cells. The results showed a potential photoprotective effect on skin cells using the lower tested concentration of 5 mg/mL: fewer γ-H2AX positive cells than the untreated control were counted through fluorescence microscopy, with a reduction in the percentage equal to 24%. The collagen assay showed a promising anti-ageing effect of the cosmetic product. The ability to promote collagen production was evaluated and shown after 24 h of treatment, with both tested concentrations and a dose-dependent increase in collagen content equal to 1078% and 1115%, respectively, compared to untreated cells.

Along with wrinkles, pigmentary changes are associated with premature photo-ageing and are its evident cutaneous manifestations. The whitening power of this serum, enriched with 30% of 3-*O*-ethyl-l-ascorbic acid in combination with 1% of lactic acid, were evaluated on reconstructed human pigmented epidermis (EPISKIN Laboratories, France). After repeated treatment for 4 days, there was a statistical reduction in the melanin content of 15.52% compared to a reference negative control. These results demonstrate another beneficial effect of this cosmetic product in association with its high biocompatibility, photoprotection, and collagen enhancing production, making the formulation useful to prevent skin modifications, ageing-related, overall.

To conclude, our study represents a promising starting point for the evaluation of C-SHOT SERUM effectiveness using different analytical approaches based on monolayer and 3D cell models. Nevertheless, further in vivo investigations will be useful for a more suitable and careful evaluation of the biological serum activity to obtain a predictive index of the in vitro data respecting the in vivo real effect of the serum.

## Figures and Tables

**Table 1:** Functional classification of ingredients contained in the serum used in the study.

Function	Ingredient
Skin conditioning	3-*O*-ethyl ascorbic acid, lactic acid
Humectant	propanediol, sodium lactate
Buffering & Keratolytic	sodium lactate
Preservative	phenoxyethanol, imidazolidinyl urea
Thickener	hydroxyethylcellulose
Chelating agent	disodium edta

**Table 2:** Technical data and batch information of maintenance and growth media.

	Maintenance Media	Growth Media
RHE (19-RHE-036)	19SMM010	19SGM026
RHPE (19-RHPE-019)	19SMM019	19SGMRHPE013

**Table 3:** Percentage changes (mean ± SD) in cell viability after 24 h of treatment with the product (*n* = 3, replicates = 3). * approximate value.

**Sample (mg/mL)**	0	0.313	0.625	1.25	2.50	5	10	20	40
**Cell Viability (%)**	100	109.78	114.20	120.52	117.04	110.91	109.60	72.89	52.18
**Standard Deviation (SD)**	0	10.86	10.71	13.69	9.89	9.31	3.29	4.30	0.93
**IC50 Value**	0.93 *								

**Table 4:** Percentage values in cell viability after treatment (42 min) and 42 h of recovery of the RHE inserts with negative control (Ctrl −), positive control (Ctrl +), and with the serum (*n* = 1, replicates = 4).

Sample	Cell Viability (%)
Ctrl −	100 ± 19.75
Ctrl +	3.17 ± 0.81
C-SHOT SERUM	114.34 ± 9.35

**Table 5:** Amount of IL-1α (IU/mL) after treatment (42 min) and 42 h of recovery of the RHE inserts with negative control (Ctrl −), positive control (Ctrl +), and the serum (*n* = 1, replicates = 4).

Sample	IL-1α (IU/mL)
Ctrl −	5.90 ± 0.65
Ctrl +	115.27 ± 0.83
C-SHOT SERUM	0.98 ± 0.17

**Table 6:** γ-H2AX positive cells (± SD) expressed as a percentage in control and positive control samples and after treatment with the product (*n* = 2, replicates = 2).

Sample	γ-H2AX Positive Cells (%)
Ctrl	6.15 ± 2.82
Ctrl +	65.70 ± 2.51
C-SHOT SERUM (5 mg/mL)	49.87 ± 5.36
C-SHOT SERUM (10 mg/mL)	52.83 ± 11.40

**Table 7:** Collagen levels expressed as a concentration (± SD) measured after treatment with tested product (*n* = 3, replicates = 2).

Sample	Collagen Levels (μg/mL)
Ctrl	0.08 ± 0.01
C-SHOT SERUM (1.25 mg/mL)	0.99 ± 0.09
C-SHOT SERUM (2.5 mg/mL)	1.24 ± 0.12

**Table 8:** Melanin quantification, expressed as μg/mL and percentage, after treatment (daily treatment for four days) of the RHPE inserts with the tested product. Negative control (Ctrl −) (*n* = 1, replicates = 4).

Sample	Melanin Content (μg/mL)	Melanin Content (%)	Reduction (%)
Ctrl −	20.02 ± 1.43	100.00 ± 8.03	-
C-SHOT SERUM	16.60 ± 1.92	82.89 ± 9.57	−17.10

## Data Availability

Data are included in the text; raw data are available from the corresponding author.
